# Creatine kinase elevation caused by a combination of fluvastatin and telmisartan in a patient heterozygous for the CYP2C9*3 and ABCC2 -24C > T variants: a case report

**DOI:** 10.1186/1756-0500-7-688

**Published:** 2014-10-03

**Authors:** Henriette E Meyer zu Schwabedissen, Werner Siegmund, Heyo K Kroemer, Jens D Rollnik

**Affiliations:** Biopharmacy, Department of Pharmaceutical Sciences, University of Basel, Basel, Switzerland; Department of Pharmacology, Ernst Moritz Arndt University of Greifswald, Greifswald, Germany; Medical Faculty, University of Göttingen, Göttingen, Germany; Institute for Neurorehabilitational Research (InFo), BDH-Clinic, Hessisch Old- endorf, Germany, Teaching Hospital of Hannover Medical School (MHH), Kragujevac, Germany

**Keywords:** Fluvastatin, OATP1B1, CYP2C9, Cytochrome P450, Telmisartan, Genetic polymorphisms, Drug transporter, Drug-drug interaction

## Abstract

**Background:**

Genetic factors as predictor of the individual outcome of drug therapy is one aim of personalized medicine approaches.

**Case presentation:**

We report a drug metabolism based analysis of genetic polymorphisms in a Caucasian patient receiving fluvastatin and telmisartan experiencing myotoxicity (myalgia and moderate creatine kinase elevation).

**Conclusions:**

The obtained findings suggest that heterocygocity of cytochrome P450 CYP2C9*3 variant in combination with multidrug resistance-associated protein MRP2 -24C > T functions as risk factor predisposing to experience drug-drug interaction combing those drugs.

## Background

Herein we report the case of a patient receiving fluvastatin for several years developing mild myotoxicity symptoms after co-administration of telmisartan, an AT1-receptor antagonist. Importantly, fluvastatin even if exhibiting a rather modest lipid-lowering effect has been widely recommended for patient receiving pharmacotherapy with high interaction potential due to the assumed low risk of pharmacokinetic interaction [1]. It was aim of this retrospective pharmacogenetic analysis to provide insight into genetic risk factors for the observed drug-drug interaction. In order to identify the predisposing factors the patient was assessed for frequently occurring single nucleotide polymorphisms of genes involved in the pharmacokinetic pathway of fluvastatin and telmisartan, respectively. Based on our current understanding fluvastatin is assumed to be taken up into the hepatocyte mediated by the hepatic uptake transporter organic anion-transporting polypeptide (OATP) OATP1B1, where it exerts its pharmacodynamic action by inhibiting the key enzyme of hepatic cholesterol synthesis the HMG-CoA reductase. Subsequent metabolism mediated by the microsomal cytochrome P450 enzymes especially the isoform CYP2C9 is the major step of drug elimination. In addition recent findings indicate that the efflux transporter BCRP (*ABCG2*) is also involved in modulating fluvastatin disposition [2, 3]. For telmisartan it is assumed that OATP1B3 is predominantly involved in hepatic uptake [4], after intrahepatic metabolism mediated by UGTs the telmisartan glucuronide is eliminated via the efflux transporters ABCB1, ABCC2 and ABCG2 [5]. In addition, Weiss and co-workers reported that telmisartan is a potent inhibitor of ABCG2 and ABCC2 mediated efflux in vitro [6]. Importantly, recent findings by Cabaleiro et al suggest that pharmacokinetics of telmisartan itself is not altered by genetic variants of CYP2C9 [7], even if telmisartan is an inhibitor of this particular enzyme [8].

Every sequential step in the process of hepatic uptake, metabolism and elimination can serve as subject of drug interaction or result in genetic variability. In this case the patient experienced myalgia accompanied by CK elevation after receiving fluvastatin and telmisartan, an angiotensin II type 1 receptor (AT1) – antagonist.

In order to elucidate the impact of common genetic variability on the observed drug interaction we determined well documented impaired function SNPs that have been associated with fluvastatin kinetics. To our knowledge this is the first case in the literature describing *in vivo* statin side effects in association with an AT1- receptor blocker.

Most AT1-blockers are metabolized by CYP2C9 [2]. Point mutations or single-nucleotide polymorphisms (SNPs) in the CYP2C9 genes have been identified. The most common coding mutations in CYP2C9 are CYP2C9*2, and CYP2C9*3 [2]. CYP2C9*2 and CYP2C9*3 differ from the wild-type CYP2C9*1 by a single point mutation: CYP2C9*2 is characterized by a 430C > T exchange in exon 3 resulting in an Arg144Cys amino acid substitution, while CYP2C9*3 is characterized by a 1075A > C exchange in exon 7 causing an I359L substitution in the catalytic site of the enzyme [2]. Individuals with the *2 or *3 variant alleles may have reduced enzyme activity [2].

## Case presentation

### DNA sample

The patient was included in a Pharmacovigilanz Study initiated by the Department of Pharmacology of the Ernst Moritz Arndt University in Greifswald. DNA sampling and subsequent genotyping of Genes involved in pharmacokinetics and -dynamics was approved by the Ethic Committee of the University Greifswald. DNA was isolated from peripheral blood cells using a QIAcube. Purity and content of DNA was assured by NanoDrop® spectrometry.

Genotyping of CYP2C9*2 (rs1799853) and CYP2C9*3 (rs1057910) variant was performed by duplex pyrosequencing. Briefly, the fragments containing the c.430C > T (CYP2C9*2) or the c.1075A > C polymorphism were amplified by duplex PCR using the following PCR primers: 5′-GTATTTTGGCCTGAAACCCATA-‵3 (CYP2C9-A2-sense) , 5′-biotin CACCCTTGGTTTTTCTCAACTC-‵3 (CYP2C9-A2-antisense), 5′-biotin TGCACGAGGTCCAGAGAT-‵3 (CYP2C9-A3-sense), and 5′-GATACTATGAATTTGGGGACTT-‵3 (CYP2C9-A2-antisense). The PCR resulting in biotinylated amplicons was performed in a 50 μl reaction volume containing 1 x reaction buffer, 0.25 μl *Platinum* Taq DNA Polymerase (invitrogen), 0.2 mM dNTPs, 1.5 mM MgCl, 40 ng template DNA and a final concentration of 200 nM of each primer. The PCR amplification started with an initial denaturation at 95°C for 15 minutes, followed by 45 cycles of denaturation at 95°C for 45 seconds, annealing at 55°C for 45 seconds, and extension at 72°C for 45 seconds, followed by a final extension step at 72°C for 5 minutes. Subsequently the amplified fragments were purified. Briefly, 40 μl of biotinylated PCR products were incubated with 5 μl streptavidin-coated sepharose beads (GE Healthcare Bio-Sciences, Munich Germany) diluted in 35 μl of PyroMark® Binding Buffer (Qiagen, Hilden Germany). After 5 minutes incubation biotinylated single stranded PCR products bound by the beads were isolated using the PyroMark® Q96 Vacuum Prep Workstation (Qiagen). After treatment with 50% ethanol, denaturation in 0.2 M sodiumhydroxid-solution and washing with 10 mM TRIS-Acetat (pH 7.6) biotinylated single strands were released into designated wells containing PyroMark® Annealing Buffer (36 μl) and 4 pmol of sequencing primers 5′-GGGAAGAGGAGCATTGAGGAC-‵3 (CYP2C9-A2 SEQ) (CYP2C9-A3 SEQ) and 5′-TGGTGGGGAGAAGGTC-‵3, followed by a 2 minutes incubation at 80°C. Subsequently, genotyping was performed using the PSQ 96MA, the PyroMark® Gold Q96 reagents, and the PSQ 96-Software (Qiagen). Genotyping of the frequently occurring polymorphisms of SLCO1B1, SLCO1B3, SLCO2B1, ABCC2 and ABCG2 was performed using commercially available TaqMan® SNP Genotyping Assays for SLCO1B1 c.521 T > C (rs4149056), SLCO1B1 c.388A > G (rs2306283), SLCO1B3 c.699A > G (rs7311358), SLCO2B1 c.935G > A (rs12422149), SLCO2B1 c.1457C > T (rs2306168), MRP2 (ABCC2), ABCC2 -24C > T (rs717620) ABCC2 c.1294G > A (rs2273697), ABCC2 c.3972C > T (rs3740066) and ABCG2 c421C > T (rs2231142) (Life Technologies GmbH, Darmstadt, Germany). After automated DNA extraction from the blood sample performed as described by the manufacturer using a QiaCube® (Qiagen, Hilden Germany) the DNA content was determined photometrically using a NanoDrop® (Peqlab, Erlangen Germany). After dilution to 10 ng/μl the DNA sample was stored at -20°C. Genotyping was performed using the pre-developed TaqMan® SNP Genotyping Assays (Applied Biosystems, Darmstadt, Germany). In detail, reactions were carried out in a 5 μl volume containing 1 μl genomic DNA, 0.25 μl Primer/Probe-Mix, 2.5 μl Genotyping Master Mix and 1.25 μl water (Applied Biosystems). Fluorescence was assessed for using the Fast Real-Time PCR system 7900 HT (Applied Biosystems) and the Sequence Detection Software SDS 2.3.

## Case report

A 39 year old male patient of Caucasian ethnicity, was treated for dyslipidemia with 40 mg/d fluvastatin, starting in January 2006. The patient had only moderately elevated cholesterol serum levels (total cholesterol 270 mg/dl; LDL cholesterol 179.8 mg/dl) but had a positive family history of cardiovascular diseases. Until June 2008, fluvastatin was combined with the cholesterol absorption inhibitor ezetimibe (10 mg/d). Although this medication was well tolerated (lipid levels as well as liver enzymes and creatine kinase (CK) are displayed in Table 1), the combination with ezetimibe was discontinued due to gastrointestinal side effects reported by the patient. During combination of ezetimibe and fluvastatin, the patient had CK levels (185-234 U/L) in the upper normal range (CK norm levels 60-175U/L). In September 2008, treatment with the AT1 blocker telmisartan (20 mg/day) was started due to borderline hypertension. After 4 weeks of treatment, the patient complained of mild myalgia, cramps und fasciculations in the legs. Subsequent testing for CK revealed an approximately two-fold increase of plasma levels (229 U/L to 439 U/L) compared to baseline. Telmisartan medication was stopped immediately and CK decreased to normal level 5 days later (145 mg/dl). Myalgia and cramps were no longer reported by the patient. Anti-hypertensive therapy was changed to candesartan.

**Table 1:** **Lab results and medication of the patient from January 2006 until November 2009**

Date	01/17/06	02/26/07	05/21/07	11/22/07	03/07/08	06/27/08	10/17/08	10/22/08	11/18/09
***Lab parameters*** **:**									
LDL [mg/dl]	179.8	74.2	71.4	74.4	-	87.6	117.8	-	137.6
HDL [mg/dl]	47.0	31.0	49.0	51.0	-	52.0	48.0	-	46.0
CK [U/l]	n.d.	215	191	223	234	229	439	145	212
GGT [U/l]	n.d.	29	27	25	22	20	25	-	36
***Medication*** **:**									
Fluvastatin	-	40 mg/d	40 mg/d	40 mg/d	40 mg/d	40 mg/d	40 mg/d	40 mg/d	40 mg/d
Ezetimibe	-	10 mg/d	10 mg/d	10 mg/d	10 mg/d	-	-	-	-
Telmisartan	-	-	-	-	-	-	20 mg/d	-	-
Candesartan	-	-	-	-	-	-	-	8 mg/d	8 mg/d

(CK norm level 60-175U/L).

**Figure 1:**
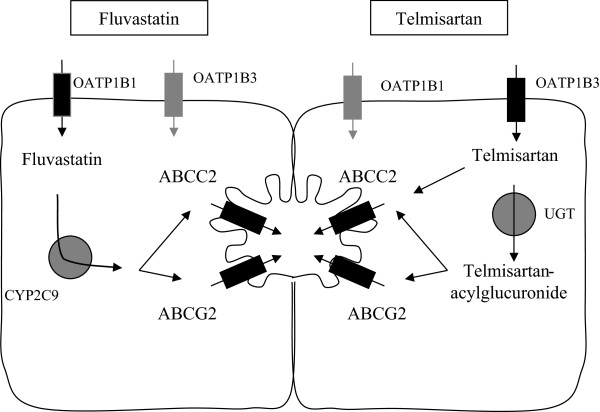
**Illustration of fluvastatin and telmisartan pharmacokinetics.** Drug transporters and metabolizing enzymes involved in the heptaocellular handling of fluvastatin and telmisartan are depicted. Transporters in black are more likely subject of drug drug interactions. UGT uridine diphosphate glucuronosyltransferase

**Figure 2:**
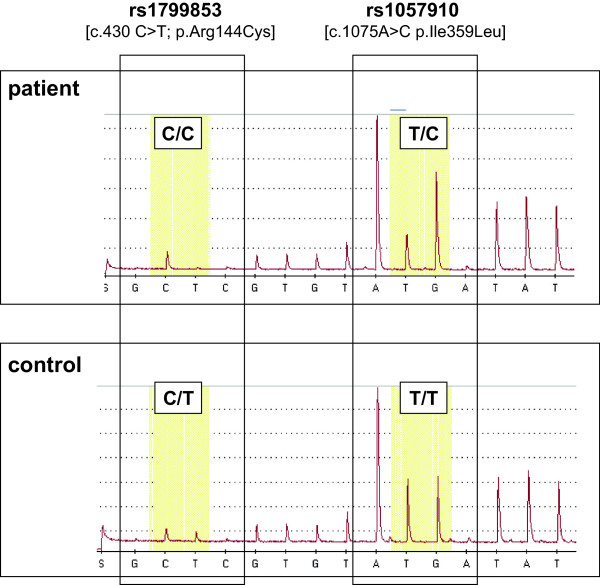
**Result of cytochrome P450 CYP2C9 genotyping of the patient in comparison to a control sample.**

Retrospective genotyping of the patient for frequently occurring single nucleotide polymorphisms (SNPs) in genes previously described to be involved in fluvastatin and telmisartan kinetics, was performed respectively (illustrated in Figure 1). As displayed in Table 2, the patient did not show genetic variability for the hepatic uptake transporter OATP1B1 (*SLCO1B1* c.388G > A (p.N130D), c.521 T > C (p.V174A)), OATP1B3 (*SLCO1B3* c.699G > A (p.M233I)), OATP2B1 (*SLCO2B1* c. 935G > A (p.R312Q), c.1457C > T (p.S486F)) and the efflux transporter ABCG2 (c.421C > A (p.Gln141Lys; rs2231142). However, genotyping of frequently occurring polymorphisms of the major metabolizing enzyme of fluvastatin namely CYP2C9 revealed that the patient is heterozygote carrier of the less frequent occurring SNP located in exon 7 (CYP2C9*3; c.1075A > C p.Ile359Leu, rs1057910), Figure 2. In addition, the patient was heterozygote for the -24C > T polymorphism located in the 5′UTR of the hepatic efflux transporter ABCC2.

**Table 2:** **Genotyping results of the patient for genes implicated in drug disposition of fluvastatin and**/**or telmisartan**

Gene	Variant	Genotype	Function in fluvastatin and telmisartan disposition
**OATP1B1**			Hepatocellular uptake of Fluvastatin
	*SLCO1B1* c.521T>C	**TT**	
	*SLCO1B1* c.388A>G	**AG**	
**OATP1B3**			Hepatic uptake of fluvastatin and telmisartan
	*SLCO1B3* c.699A>G	**AA**	
**OATP2B1**			Cellular uptake of telmisartan.
	*SLCO2B1* c. 935G>A	**GG**	
	*SLCO2B1* c.1457C>T	**CC**	
**MRP2** **(ABCC2)**			Biliary elimination of telmisartan acylglucuronide
	*ABCC2* -24C>T	**CT**	
	*ABCC2* c.1294G>A	**GG**	
	*ABCC2* c.3972C>T	**CC**	
**BCRP** **(ABCG2)**			Biliary elimination of telmisartan acylglucuronide
	*ABCG2 c421C*>*T*	**CC**	
**CYP2C9** * **3**			Biliary elimination of fluvastatin.
	*CYP2C9 c.1075A*>*C*	**CT**	

## Conclusions

In this report we describe the retrospective genetic analysis of a patient who experienced mild myalgia and CK increase after co-administration of fluvastatin with the AT1- receptor antagonist telmisartan. Findings suggest that that genetic variability of CYP2C9 in addition to the ABCC2 (-24C > T) variant might be a risk factor for developing drug-drug interactions combining fluvastatin and telmisartan.

Like other exogenous compounds, statin therapy harbours the risk of rare but significant adverse reactions targeting organs such as muscle (myotoxicity) or liver (transaminase elevation). In particular, the risk of rhabdomyolysis which can lead to renal failure and death has to be addressed. The finding of a recent report showing that a functional polymorphism in the hepatic uptake transporter OATP1B1 (*SLCO1B*1) is the only predictor of simvastatin induced myotoxicity [9] has given rise to several studies focussing on effects of this particular polymorphism on statin disposition [10].

In general, fluvastatin undergoes extensive fist pass metabolism resulting in a bioavailability of only 19-29%. In addition, the majority of fluvastatin is eliminated as metabolites into the bile, and it has been suggested that CYP2C9 accounts for 50-80% of fluvastatin metabolism, whereas CYP2C8 and CYP3A4 play only a minor role. In line with this finding it has been shown that impaired function alleles of CYP2C9 are associated with changes in fluvastatin disposition and efficacy in healthy volunteers [11]. In particular, CYP2C9 exhibits commonly occurring SNPs, namely the CYP2C9*2 (c.430 C > T; p.Arg144Cys; rs1799853) variant, which is frequent among Caucasians with approximately 1% of the population being homozygous carriers and 22% heterozygous, whereas the corresponding figures for the CYP2C9*3 (c.1075A > C p.Ile359Leu, rs1057910) allele in a Caucasian population are much lower with only 0.4% and 15%, respectively [6]. Especially the CYP2C9*3 allele, which is associated with the nucleotide exchange (c.1075A > C) in exon 7 and results in an amino acid exchange in the catalytic site of the enzyme has been previously shown to result in impaired metabolism of substrate drugs [12]. However, the effect of genetic variants on catalytic activity and the clinical impact seems to be substrate specific.

Genotyping for the above described polymorphisms in CYP2C9 revealed that the patient was heterozygous for CYP2C9*3 allele, suggesting that the patient carries a predisposition for higher fluvastatin plasma levels, which could contribute to increased susceptibility for adverse side effects. However, the history of the patient shows that the CYP2C9*3 variant alone was not the only factor inducing myalgia and CK elevation, which began after co-administration of telmisartan. This is in accordance with the previously described lack of association of genetic variants of CYP2C9 variants with fluvastatin induced myotoxicity. However, it should be noted that only one patient was carrier of the CYP2C9*3 variant in this report [13].

Based on the current understanding of telmisartan pharmacokinetics, the drug-drug interaction described in our patient could involve additional mechanisms especially as telmisartan is only a moderate CYP2C9 inhibitor [7, 8]. However, in individuals harbouring a low function allele of CYP2C9 in heterocygocity, the inhibitory capacity might be of clinical significance. Taken together, the importance of otherwise non-significant pharmacokinetic drug-drug interaction in the presence of pharmacokinetic variant alleles like CYP2C9*3 merits further discussion.

However, the majority of telmisartan is assumed to be eliminated hepatically in a glucuronidated form mediated by the efflux transporters MRP2 (ABCC2), BCRP (ABCG2) and MDR1 (ABCB1) [8]. Testing the influence of frequently occurring SNPs in ABCB1 and ABCC2 revealed an impact of the ABCC2 -24C > T variant on telmisartan disposition [9, 10]. Considering the inhibitory capacity of telmisartan on CYP2C9 mediated catalysis, which had been identified *in vitro* by Kamiyama and co-workers (IC_50_ 41.9 ± 15.1 μM)., the identified heterocygocity of the patient for the MRP2 -24C > T allele gives reason for the speculation of this polymorphism being involved in the susceptibility of the herein described drug-drug interaction. However, Kamiyama and co-workers who conducted the *in vitro* study, suggested that due to the high Ki in association with relatively low free plasma levels of telmisartan would be unlikely to exert clinically relevant inhibition of CYP2C9 [8]. In accordance with those findings are results from an *in vivo* study showing that telmisartan did not influence the plasma levels and efficacy (INR) of warfarin another CYP2C9 substrate [14].

However, we assume that the prevalence of both predisposing factors reduced hepatic elimination of telmisartan in association with reduced hepatic metabolism of fluvastatin, resulted in the herein described drug-drug interaction. This report further strengthens the hypothesis that the success of “personalized medicine” in terms of genotype driven risk stratifications are not sufficiently supported by monogenetic pharmacological analyses, which are commonly conducted [15]. This case illustrates that a combination of both – pharmacogenomics and the knowledge of drug-drug interactions – could help to achieve better clinical outcomes by personalized medicine.

## Consent

Written informed consent was obtained from the patient for publication of this Case Report and any accompanying images. A copy of the written consent is available for review by the Editor-in-Chief of this journal.

## Abbreviations

AT1: Angiotensin II receptor type 1
BCRP: Breast cancer resistance protein
CK: Creatine kinase
CYP: Cytochrome P450
DNA: Deoxyribonucleic acid
HMG-CoA: 3-hydroxy-3-methylglutaryl-coenzyme A
INR: International normalized ratio
MRP: Multi-resistance protein
OATP: Organic anion-transporting polypeptide
PCR: Polymerase chain reaction
SNP: Single-nucleotide polymorphism.
